# Tax impairs DNA replication forks and increases DNA breaks in specific oncogenic genome regions

**DOI:** 10.1186/1476-4598-13-205

**Published:** 2014-09-04

**Authors:** Hassiba Chaib-Mezrag, Delphine Lemaçon, Hélène Fontaine, Marcia Bellon, Xue Tao Bai, Marjorie Drac, Arnaud Coquelle, Christophe Nicot

**Affiliations:** Department of Pathology and Laboratory Medicine, University of Kansas Medical Center, 3901 Rainbow Boulevard, Kansas City, KS 66160 USA; IRCM, Institut de Recherche en Cancérologie de Montpellier, Montpellier, F-34298 France; INSERM, U896, Montpellier, F-34298 France; Université Montpellier 1, Montpellier, F-34298 France; Institut régional du Cancer Montpellier, Montpellier, F-34298 France; DNA Combing Facility, Institut de Génétique Moléculaire, CNRS UMR5535 & BioCampus Montpellier (UMS3426), 1919 route de Mende, Montpellier cedex 5, 34293 France

## Abstract

**Background:**

Human T-cell leukemia virus type 1 (HTLV-I) is a human retrovirus associated with adult T-cell leukemia (ATL), an aggressive CD4 T-cell proliferative disease with dismal prognosis. The long latency preceding the development of the disease and the low incidence suggests that the virus itself is not sufficient for transformation and that genetic defects are required to create a permissive environment for leukemia. In fact, ATL cells are characterized by profound genetic modifications including structural and numerical chromosome alterations.

**Results:**

In this study we used molecular combing techniques to study the effect of the oncoprotein Tax on DNA replication. We found that replication forks have difficulties replicating complex DNA, fork progression is slower, and they pause or stall more frequently in the presence of Tax expression. Our results also show that Tax-associated replication defects are partially compensated by an increase in the firing of back-up origins. Consistent with these effects of Tax on DNA replication, an increase in double strand DNA breaks (DDSB) was seen in Tax expressing cells. Tax-mediated increases in DDSBs were associated with the ability of Tax to activate NF-kB and to stimulate intracellular nitric oxide production. We also demonstrated a reduced expression of human translesion synthesis (TLS) DNA polymerases Pol-H and Pol-K in HTLV-I-transformed T cells and ATL cells. This was associated with an increase in DNA breaks induced by Tax at specific genome regions, such as the c-Myc and the Bcl-2 major breakpoints. Consistent with the notion that the non-homologous end joining (NHEJ) pathway is hyperactive in HTLV-I-transformed cells, we found that inhibition of the NHEJ pathway induces significant killing of HTLV-I transformed cells and patient-derived leukemic ATL cells.

**Conclusion:**

Our results suggest that, replication problems increase genetic instability in HTLV-I-transformed cells. As a result, abuse of NHEJ and a defective homologous repair (HR) DNA repair pathway can be targeted as a new therapeutic approach for the treatment of adult T-cell leukemia.

## Introduction

During DNA synthesis, replication forks repeatedly come across obstacles that impede their progression. Arrested forks are very unstable and have to be restarted promptly in order to prevent the formation of DDSB and genome instability [1–3]. In normal cells, a few DDSB foci can be observed during replication of DNA in the S phase. These breaks are generally quickly repaired and the cell proceeds with division. Some oncogenes increase the rate of replication fork stalling, which facilitates chromosome rearrangement at common fragile sites in precancerous lesions and increases the transformation rate [4]. Other oncogenes increase the formation of DDSBs or interfere with the DNA repair machinery to promote transformation. DDSBs are the most dangerous form of DNA damage, because if incorrectly repaired, they cause problems for transcription, replication, and chromosome segregation [5–7].

HTLV-I-associated ATL has very limited therapeutic options and the projected 4-year survival rates for acute- and lymphoma-type ATL patients stand at 5 and 5.7%, respectively [8, 9]. Development of the disease usually follows a long latency period during which limited expression of viral genes can be detected and viremia is absent. Infected cells evade host immune clearance through the combined action of p12 and p30 [10]. Persistence and expansion of the provirus mostly occurs by cellular division leading to clonal expansion of infected cells [11]. In contrast to other onco-retroviruses, HTLV-I controls its own latency by expressing the p30 viral protein [12, 13]. Interestingly, this characteristic is not shared by HTLV-II [13]. The viral Tax protein has oncogenic properties and can immortalize human primary T cells [14], transform fibroblasts [15], and lead to various tumors in transgenic mouse models [16–19]. Numerous studies have demonstrated that Tax alters cell cycle checkpoints, prevents apoptosis, and inhibits DNA repair pathways [20–25]. In addition, Tax favors long term proliferation and survival of infected cells by stimulating telomerase expression [26, 27]. During the *in vivo* expansion of ATL cells, the expression of Tax progressively decreases and is compensated by accumulated mutations in cellular genes and constitutive activation of signaling pathways. We have previously shown that HTLV-I transformed cells have a higher than normal basal level of phosphorylated ATM (S1981) and p-H2AX, suggesting continuous formation of DDSBs [28]. Dual staining for γ-H2AX and BrDU incorporation, which marks DNA breaks in S phase, demonstrated that γ-H2AX foci were mostly detected in Tax-expressing cells with replicating DNA [29]. These findings were further confirmed by staining for γ-H2AX and Cyclin A, a marker of cells in S phase. The cells that stained positive for γ-H2AX were also positive for Cyclin A. Finally, similar results were also obtained with γ-H2AX and PCNA (Proliferating cell nuclear antigen), for which a punctuated signal is indicative of cells in S phase. These studies reveal a mutagenic activity associated with Tax expression. Moreover, we have recently demonstrated that Tax inhibited the HR repair pathway, thereby creating a “mutator phenotype” [29]. However, how Tax increases DDSBs during DNA replication and the biological consequences of the Tax-induced DDSBs remain largely unknown.

In this study we use molecular combing techniques to study the effect of HTLV-I Tax on DNA replication. We use cells that constitutively express Tax as well as cells stably transfected with an inducible Tax expression vector to check for potential cell adaptation. Our results demonstrate that replication forks are generally slower and stall more often in cells expressing Tax. The cellular response to Tax expression translated into an increase in the firing of back-up origins of replication. These observations are consistent with the notion that dormant replication origins fire in response to replication issues and generally lead to an increase in DDSBs. Our results also demonstrate that Tax does not directly provoke DNA breaks but rather that these DDSBs result from the faulty activation of the HR pathway, which is inhibited by Tax. We further demonstrate that in HTLV-I-transformed and Tax-expressing cells the expression of TLS DNA polymerases Pol-H and Pol-K are significantly reduced, further adding stress on replication of complex DNA structures and stalled replication forks. We demonstrate that the expression of chemoresistant genes Pol-H and Pol-K is down-regulated in HTLV-I- and ATL-transformed cells. Importantly, we also found that Tax expression increases DNA breaks associated with non-B-DNA conformation chromatin at the c-Myc promoter and the Bcl-2 major break point. Finally, inhibition of the NHEJ DNA repair pathway by NU7026 induces significant cell death of HTLV-I-transformed and patient leukemic cells, suggesting that drugs targeting the NHEJ pathway might offer new therapeutic options for patients with ATL.

## Results

### Replication forks are slower in the presence of the HTLV-I Tax oncoprotein

**Figure 1:**
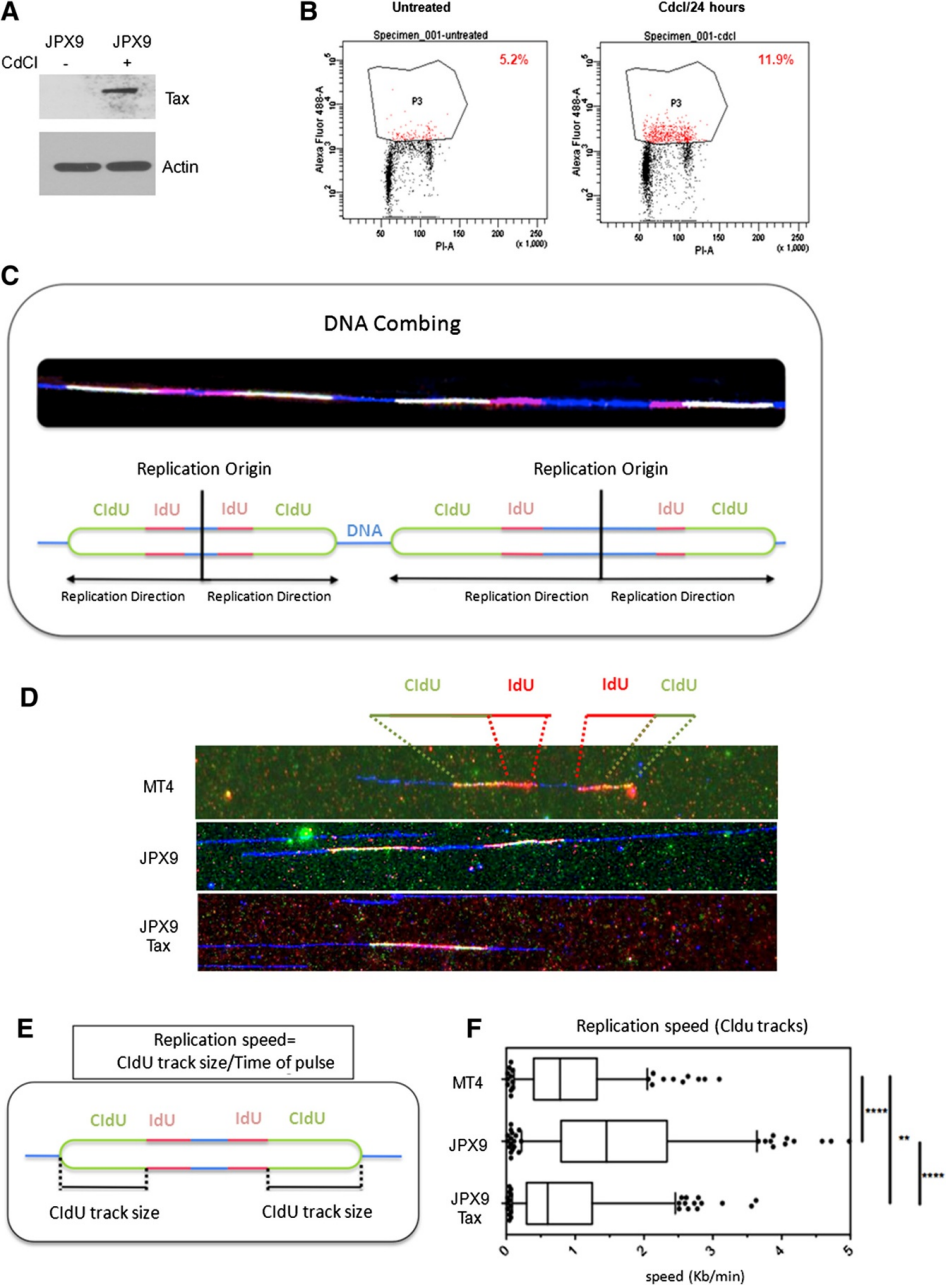
**Replication forks are slower in the presence of the HTLV-I Tax oncoprotein. (A)** HTLV-I Tax expression was confirmed after induction with Cadmium chloride (CdCl) in Tax inducible Jurkat, JPX9 cells. **(B)**. Induction of Tax expression was associated with an increase in DNA breaks detected by FACS analyses of p-H2AX. **(C)** Schematic representation of a single molecule analysis of DNA replication by molecular combing. **(D)** MT4, JPX9 and JPX9 Tax+ cells were pulse-labelled for 15 min with IdU then 15 min with CldU, and fibers were stretched by DNA combing. Blue: DNA. Red: IdU. Green: CldU. An example of a fiber representative for each condition is shown. **(E)** Replication fork speed analysis. The lengths of CldU tracks (ImageJ) were measured and divided by the length of the pulse (15 min). **(F)** Box and whisker plots: Box: 25–75 percentile range and Whiskers: 5–95 percentile range. More than 300 values coming from 3 independent experiments were compiled. Statistical analysis was performed using the non-parametric Mann–Whitney rank sum test. ns: not significant. *: p < 0.05; **: p < 0.01; ****: p < 0.0001.

The aim of this study is to investigate the effect of Tax on DNA replication. To this end we utilized HTLV-I-transformed MT4 cells (with constitutive expression of Tax) and a Jurkat cell line (JPX9) that is stably transfected with an inducible Tax-expressing vector (Figure 1A). Induction of Tax expression in Jurkat cells was associated with DDSBs and increased p-H2AX (Figure 1B). For clarity, induced JPX9 cells will be labeled JPX9 Tax+ hereafter. Using molecular combing techniques, we first asked whether expression of Tax leads to replication defects (Figure 1C). JPX9, JPX9 Tax+, and MT4 cells were pulse-labeled with two nucleotide analogs, IdU (5-iododeoxyuridine) and CldU (5-chlorodeoxyuridine), which incorporate into newly synthesized DNA. To visualize tracks of replication by DNA combing, IdU and CldU were revealed by specific fluorescent markers, red and green, respectively (Figure 1C and 1D). IdU (first pulse) is incorporated before CldU (second pulse) and allows us to determine the direction of replication fork progression and the presence of active replication origins.

Then we determined fork speed by measuring the length of CldU tracks, only when they followed an IdU track (Figure 1E and 1F). This allows us to focus only on forks that are currently active when the cells received the CldU. To obtain fork speed, the length of the track was divided by the length of the pulse (15 min). Analysis of multiple independent experiments revealed that the distance covered by individual forks during the pulse was significantly shorter and slower in the Tax-expressing cells, JPX9 Tax + and MT4, when compared to JPX9 control cells (Figure 1F). We also showed that fork speed was slower in Tax expressing cells, compared to non-Tax expressing cells. Forks were slightly slower following induced Tax expression compared to constitutive Tax expression, presumably because following induction of Tax expression, the cells are faced with replication problems they do not usually encounter and to which they are not adapted.

### Tax impedes DNA replication fork progression

**Figure 2:**
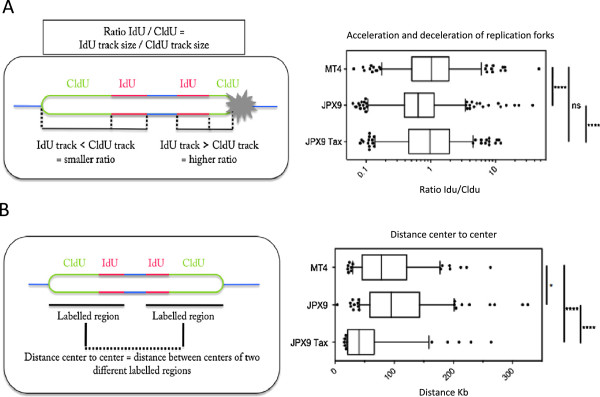
**Tax blocks DNA replication fork progression. (A-B)** Box and whisker plots. Box: 25–75 percentile range. Whiskers: 5–95 percentile range. More than 300 values coming from 3 independent experiments were compiled. Statistical analysis was performed using the non-parametric Mann–Whitney rank sum test. ns: not significant. *: p < 0.05; **: p < 0.01; ****: p < 0.0001. **(A)**: Measurement of acceleration and deceleration of replication forks, IdU/CldU ratio. **(B)**: Analyses of the center-to-center distance which reflects the distance between two replication origins and is indicative of origin firing frequency.

We next characterized fork progression in the different conditions by determining the IdU/CldU ratio (Figure 2A). This ratio allows us to determine whether replication forks progress continuously or encounter obstacles that can slow or stop them: in this latter case, the length of the CldU track should be shorter than the IdU track, leading to an increased ratio. We show that Tax-expressing cells have a higher ratio than non-Tax-expressing cells, meaning that, for each single replication fork, the more they replicate DNA, the more problems they accumulate. The shorter labeled tracks observed in MT4 and JPX9 Tax+-expressing cells compared to JPX9 cells could be due to slower forks or to an increase in fork stalling. To discriminate between these two possibilities, the progression of sister replication forks was analyzed by DNA combing. In normal cells, sister forks progressed at a similar rate from a given origin and generated symmetrical patterns of IdU/CldU incorporation. We analyzed the ratio of the longest to the shortest IdU signals to reveal any increase in fork asymmetry in Tax-expressing cells relative to controls. Our results showed that sister forks did not progress at a similar rate from a given origin and generated asymmetrical patterns of IdU/CldU incorporation. Analysis of the ratio of the longest to the shortest IdU signals for each pair of sister replication forks also revealed an increase in fork asymmetry. These results indicate that replication forks are not only slower, but also pause and stall more frequently in the presence of Tax expression. These results suggest that Tax expressing cells have greater difficulties in progressing through complex DNA structures, which may result in replication fork fallout. This is consistent with our previously reported increased formation of DDSBs observed in S phase in Tax expressing cells.

### Tax increases origin firing by activating back-up origins

Since origins of replication are at the middle of IdU/CldU incorporation signals, the initiation rate can be estimated by center-to-center distances between tracks (Figure 2B). Shorter distances are indicative of an increase in the initiation rates or origin firing. We measured the center to center distance (the distance between two tracks on the same fiber), which reflects the distance between two replication origins and is indicative of origin firing frequency (Figure 2B). Again, we show that this distance is different between Tax expressing and non-expressing cells. Since this distance is shorter in Tax expressing cells, these cells have an increased initiation rate, by activating back-up origins. These data are consistent with other studies showing that dormant replication origins fire in response to replication issues. Again, this phenotype is more prominent following induced expression of Tax (JPX9 Tax+) as opposed to constitutive Tax expression (MT4). We think that MT4 cells have adapted to constitutive expression of Tax. Our data is in agreement with the previously reported effects of Tax on the minichromosome maintenance complex and increased origin firing [30]. Our results show that Tax induction leads to multiple replication defects, partly compensated by activation of back-up origins. A summary of these data and statistically significant differences between MT-4 and JPX-9, MT-4 and Jurkat and JPX9 and Jurkat cells are summarized in Table 1 and Table 2.

**Table 1:** **Tax impedes DNA replication fork progression**

Median value	MT4	JPX9	JPX9 Tax
Replication speed on CldU tracks (kb/min)	0,78	1,46	0,60
Ratio IdU/CldU	1,02	0,63	0,97
Distance center-to-center (kb)	78,50	94,98	40,63

**Table 2:** **Tax increases origin firing by activating back-up origins**

P Values	MT4 vs JPX9	JPX9 vs JPX9 Tax	MT4 vs JPX9 Tax
Replication speed on CldU tracks	< 0,0001	< 0,0001	0,0297
Ratio IdU/CldU	< 0,0001	< 0,0001	0,397
Distance center-to-center	0,0442	< 0,0001	< 0,0001

### Tax-mediated increases in NO production promote the accumulation of DDSBs during DNA replication

**Figure 3:**
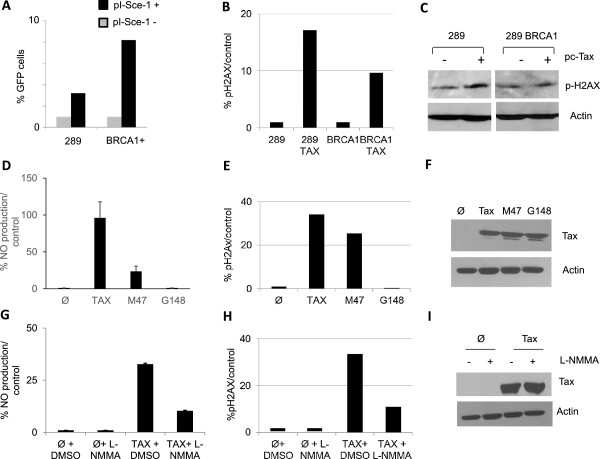
**Tax expression induces DDSBs by stimulating intracellular NO.** HR activity **(A)** is reduced in 289 cells when compared to BRCA1 cells. Tax triggers H2AX phosphorylation in BRCA1-deficient cell. The 289 and BRCA1 cells were infected with GFP or Tax-IRES GFP-expressing virus during 48 hours. 293T cells were transfected with 1.5 μg DR-GFP and 0.5 μg pSCE I expression vector or empty vector. The percentage of GFP+ cells was estimated by FACS. **(B)** Tax triggers H2AX phosphorylation in BRCA1-deficient cell. The 289 and BRCA1 cells were infected with GFP or Tax-IRES GFP-expressing virus during 48 hours. The cells were analyzed **(B)** by Flow cytometry and **(C)** by western blot. **(D-F)** Tax and M47 Tax mutant stimulate NO production and trigger H2AX phosphorylation. 293T cells were transfected with 2 μg of empty vector (Ø), Tax, or Tax mutants (M47 and G148V). At 72 h, cell culture supernatant was harvested and tested for NO production **(D)**, and cells were further analyzed by FACS for ɣ-H2AX quantification **(E)** and by western blot for Tax expression **(F)**. **(G-I)** The NOS inhibitor L-NMMA blocks NO production and DDSBs induced by Tax. 293T cells were transfected with the empty vector (Ø), Tax, or Tax mutants (M47 and G148V) during 24 h and treated with DMSO or 30 μM L-NMMA for 48 h. Cell culture supernatant was harvested and tested for NO production **(G)**. Cells were analyzed by FACS for ɣ-H2AX quantification **(H)** and western blot to confirm Tax expression **(I)**.

Our previous data demonstrated that Tax-associated DDSBs occur mainly during replication of DNA in the S phase. Hence, it is difficult to assess whether these DDSBs are induced directly by Tax or if they result from replication-associated breaks and accumulate as a result of inefficient HR-mediated repair when Tax is present. We reason that if we express Tax in a cell line already deficient in HR repair, we will then be able to assess the importance of replication-associated breaks independently of HR. Reports suggest that BRCA1-deficient cells are largely deficient in the HR repair pathway [31].Therefore, we used BRCA1−/− UWB1.289 cells (289 thereafter) and UWB1.289BRCA1, the counterpart cell line reconstituted with BRCA1 expression to test our hypothesis. We first confirmed reduced HR activity in 289 cells when compared to UWB1.289BRCA1 cells by using a previously described HR-GFP reporter assays [32] and FACS analyses. In this assay, the Sce1 restriction enzyme induces a single cleavage site in the GFP vector. Upon repair, a GFP signal is produced if the repair has occurred through the HR repair pathway. Our results first confirmed that 289 cells are indeed deficient in the HR repair pathway as shown by a significantly decreased GFP signal when compared to BRCA1-reconstituted cells (Figure 3A). We hypothesized that if Tax is able to induce breaks directly, independently from its inhibition of HR, expression of Tax in 289 cells will be associated with an increase in γ-H2AX. On the other hand, if DDSBs associated with Tax expression are indirect, and only the result of Tax-inhibition of HR during DNA replication associated breaks, expression of Tax in HR-deficient cells will not be associated with an increase in DDSBs, as detected by γ-H2AX foci. Cells were transduced with Tax-IRES GFP-expressing virus. The percentage of cells transduced was fairly equivalent for both lines 289 and 289BRCA1 cell lines as evaluated by GFP expression and FACS analyses (data not shown). We then assessed the presence of DNA breaks in each cell line in the absence of Tax or forty-eight hours after transduction with Tax-IRES GFP-expressing vector. Our results demonstrated an increase in p-H2AX between 289 and 289 BRCA1 cells (Figure 3B). These results suggest that Tax does induce DDSBs independently from its inhibitory effect of HR. Consistent with these results, western blot assays revealed a significant increase in p-H2AX only in 289 cells in the presence of Tax (Figure 3C). Since previous studies have suggested that Tax can increase the expression of iNOS in monocyte U937 cells and nitric oxide (NO) has been linked to DDSBs, we tested Tax and Tax mutants for their ability to stimulate nitric oxide (NO) production. We used Tax and Tax mutants able to stimulate either the NF-kB or the CREB pathway, M47 and G148V, respectively. Our results showed that both wild-type Tax and the Tax mutant M47 can increase NO production while the Tax mutant G148V did not (Figure 3D). Consistent with the notion that NO has been shown to produce DNA breaks at replication sites, expression of Tax and the Tax mutant M47 were associated with DDSBs, as shown by an increase in p-H2AX expression (Figure 3E). Expression levels of Tax and Tax mutants were fairly equivalent (Figure 3 F). Interestingly, the effect of Tax and Tax mutants on DDSBs correlated with their ability to activate the NF-kB pathway and to stimulate NO production inasmuch as Tax G148V was unable to produce NO and did not create DDSBs. In addition to NF-kB, other pathways activated by Tax but not the M47 mutant may be responsible for the lower NO activation seen in Figure 3D. For instance Tax-mediated AP-1 activation is not shared by the M47 mutant [33].To further demonstrate a direct link between Tax-mediated NO production and the formation of DDSBs, we treated Tax expressing cells with a specific NO inhibitor, N-Monomethyl-L-arginin (LNMMA). As expected, treatment with LNMMA greatly reduced the ability of Tax to stimulate NO production (Figure 3G). Consistent with our hypothesis, decreased Tax-mediated NO production was associated with a decrease in DDSB formation (Figure 3H), while Tax expression was not affected (Figure 3I). Altogether our results demonstrate that Tax directly induces DDSB at replication sites by stimulating intracellular NO production and impairing DNA replication forks.

### Reduced expression of human translesion synthesis (TLS) DNA polymerases in HTLV-I-transformed and Tax-expressing cells

**Figure 4:**
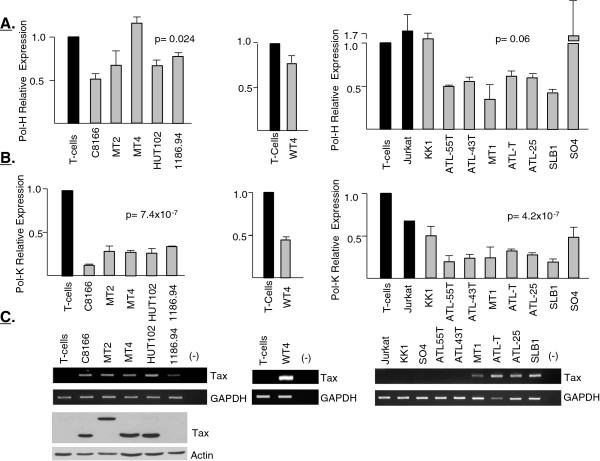
**Reduced expression of Pol-H (A) and Pol-K (B) in HTLV-1 cell.** Real-time PCR was performed in duplicate and samples were normalized to GAPDH expression. Fold change was calculated by comparing values with T cells (sorted from healthy donor) and/or Jurkat. **(C)** Viral *tax* mRNA expression was demonstrated by RT-PCR. Tax protein expression was detected by immunoblots in HTLV-I-transformed cell lines *in vitro*.

Cells are constantly exposed to DNA damaging agents. Lesions induce activation of checkpoints and G1 arrest to allow repair before the cells proceed to S phase and DNA replication. Tax-expressing cells bear a high rate of DDSBs and have multiple defects in cell cycle checkpoints. When DNA lesions are not repaired before DNA replication is initiated, it can block the replication machinery and lead to cell cycle arrest and apoptosis. To bypass these blocks and proceed with DNA replication, specialized translesion synthesis (TLS) DNA polymerases of the γ-family are recruited and allow error-prone replication through damaged DNA [34]. Expression of TLSs in HTLV-I transformed cells has not been previously investigated. TLS expression is mainly controlled by the tumor suppressor p53 and by p21WAF [35, 36]. In cells where p53 or p21WAF are inactivated, TLS expression is deregulated, leading to unrestrained mutagenic bypass of DNA lesions, accumulation of mutations, and increased drug resistance of cancer cells. In addition, a loss in the expression of TLS’s Pol-H or Pol-K, expression also results in increased DDSBs and genome instability. Our results showed a significant reduction in the expression of Pol-H and Pol-K in HTLV-I-transformed cells (Figure 4A and 4B). We also found that both Tax-negative ATL-derived cell lines and Tax-expressing ATL-derived cell lines had significantly reduced expression of Pol-K and Pol-H (Figure 4A, 4B and 4C).

Although transient expression of Tax in Jurkat cells was clearly associated with DNA breaks and increased p-H2AX, we could not demonstrate a down-regulation of Pol-H expression and Tax had a small but reproducible inhibitory effect of about 15% on Pol-K expression (data not shown). In contrast, down regulation of Pol-H and Pol-K was readily observed in the Tax-immortalized primary T cell line, WT4 (Figure 4A and 4B). These results suggest that chronic expression of Tax may be responsible for adaptation and reduced expression of Pol-H and Pol-K in HTLV-I- and ATL-transformed cells and warrants further studies.

### Tax expression increases DNA breaks associated with non-B-DNA conformation chromatin at the c-Myc promoter and the Bcl-2 major break point

**Figure 5:**
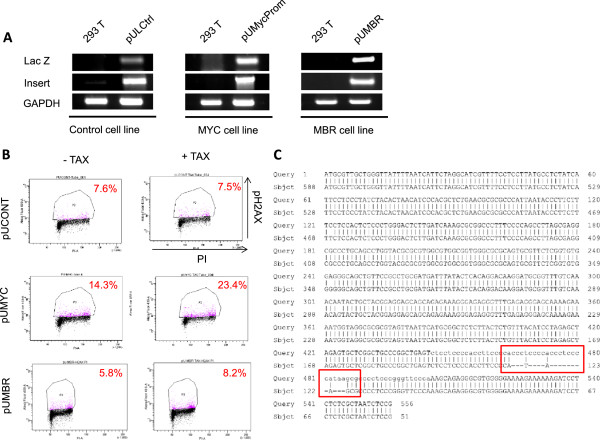
**Tax expression increases DNA breaks and rearrangement in a human c-MYC promoter region rich in non-B-DNA forming sequences. (A)** Establishment of stable cell lines containing a non-DNA sequence control from the GAPDH gene (pULCtrl), a c-MYC promoter region (pUMyc), and a BCL-2 gene major break region (pUMBR) as described in the Material and Methods. **(B)** pUCONT, pUMYC and pUMBR cell lines were infected with HRCMV or HRCMVTax virus for 48 h and analyzed by Flow cytometry to quantify ɣ-H2AX. Scatter plots are represented with ɣ-H2AX intensity on the y axis and propidium iodide intensity on the x axis. The cell populations used for the measurement of the ɣ-H2AX-positive cells were enclosed by rectangles. **(C)** Tax induces a small scale deletion in the c-MYC promoter. After 48 h infection by HRCMV or HRCMVTax, the genomic DNA was isolated from the pUMyc cells and subjected to PCR to amplify c-MYC promoter region. The PCR product was cloned into pGEM-T Easy vector and the inserts were verified by DNA sequencing.

Endogenous sources of interference with replication fork progression exist within complex DNA sequences. These have the potential to adopt non-B-DNA conformations and are abundant in chromosomal regions that are prone to rearrangements, such as the major breakage hotspots found in c-Myc or sequences that form G quadruplex DNA. These DNA structures are physiological substrates of both Pol-H and Pol-K, which are required when replication forks are challenged by non-B-DNA to facilitate replication fork reactivation and/or avoid fork collapses and DDSB induction. Since loss of Pol-H or Pol-K expression results in increased DDSBs, down-regulation of these TLS DNA polymerases could be a contributing factor in Tax-induced DNA breaks in S phase. To study the effect of Tax expression on non-B-DNA conformations, we constructed different cell lines with stable integration of either a plasmid containing G-rich sequences from the human c-Myc promoter region (pUMycProm) or a sequence from the human Bcl-2 major break point region (pUMBR) into their genome (Figure 5A). As a control we also established a cell line with stable integration of B-DNA sequence from the GAPDH gene (pUCONT).

To investigate the effects of Tax on non-B-DNA structures, we generated high titer Tax-expressing virus (HRCMVTax) to reach high efficiency of transduction. pUMYC, pUMBR and pUCONT cell lines were infected with HRCMVTax and analyzed after 48 hours for DNA breaks by immuno-detection of p-H2AX and quantification by FACS. Our results showed a reproducible increase in DNA breaks of about 2.5 and 9% for pUMBR and pUMYC (compared with and without Tax expression), respectively (Figure 5B). Such a percentage increase in p-H2AX is consistent with previous studies performed with siRNA knock-down of Pol-H and Pol-K [37]. Interestingly, we found that the effect of Tax was more pronounced at the c-Myc promoter, suggesting that Tax may preferentially target particular genomic regions. Sequencing analyses revealed internal deletions at the c-Myc promoter in the presence of Tax in 8% of clones tested (Figure 5C).

### NHEJ inhibition induces cell death in HTLV-I-transformed and ATL cells

**Figure 6:**
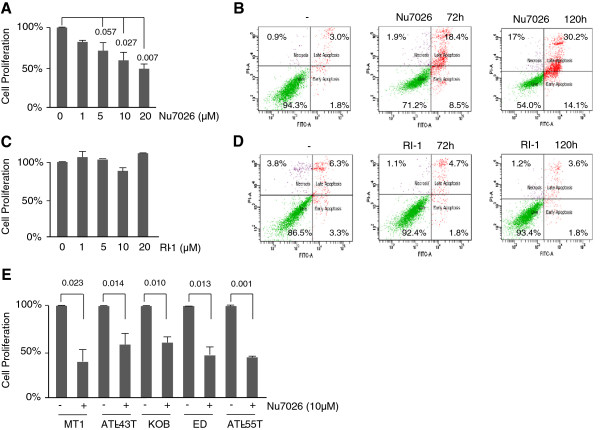
**NU7026 inhibits growth and induces apoptosis in HTLV-I cell lines, but not RI-1. (A)** MT4 cells were treated with increasing concentrations of NU7026. After 3 days of treatment, cells were stained with XTT and measurements were quantified at OD: 450 nm. The results represent the means of 2 separate experiments + SD. **(B)** MT4 cells were treated with NU7026 (10 μM). After 3 and 5 days of treatment, cells were stained with Annexin V and PI, and analyzed by flow cytometry. **(C)** MT4 cells were treated with an increasing concentration of RI-1. After 3 days of treatment, cells were stained with XTT and measurements were quantified at OD: 450 nm. The results represent the means of 2 separate experiments + SD. **(D)** MT4 cells were treated with RI-1 (20 μM). After 3 and 5 days of treatment, cells were stained with Annexin V and PI, and analyzed by flow cytometry. **(E)** ATL cell lines were treated with NU7026 (10 μM) for 3 days. Then, cells were stained with XTT and measurements were quantified at OD: 450 nm. The results represent the means of 2 separate experiments + SD.

Our previous studies demonstrated that Ku80 and DNA-PK specifically co-localized with γ-H2AX foci on DNA breaks in Tax-expressing cells, while RAD51 foci, a marker for HR repair, co-localized with γ-H2AX in control cells but not in Tax-expressing cells. We further demonstrated that in Tax-expressing cells, DDSBs are repaired mostly in an HR-independent manner through the activation of the NHEJ pathway. We hypothesized that the faulty activation of the NHEJ repair pathway during S phase may cause HTLV-I-transformed cells to become resistant to chemotherapeutic agents. We then tested the effect of NU7026, a specific DNA-PK and NHEJ inhibitor. The use of NU7026 had a significant inhibitory effect on the proliferation of HTLV-I-transformed MT4 cells as shown by XTT assays (Figure 6A). These data are consistent with previous reports showing a G2/M arrest after treatment with NU7026 and the presence of a functional G2/M checkpoint in HTLV-I-transformed cells.

We next tested a RAD 51 inhibitor of the HR repair pathway, RI-1. RAD51 was found to be overexpressed in many tumors and transformed cell lines and increased RAD51 expression correlates with resistance to chemotherapies and radiotherapies [38]. Previous studies have shown that RI-1 (20 μM) completely inhibited the formation of DNA damage-induced RAD51 foci in immortalized human fibroblasts and more than 90% of HR DNA repair [39]. Higher concentrations of RI-1 have also been shown to inhibit single-strand annealing (SSA) [39]. These observations may be important because SSA repair efficiency is known to be increased when RAD51 functions are disrupted, which has been shown to be the case for HTLV-I. It is important to evaluate which of the DNA repair pathways are essential for survival of HTLV-I-transformed cells, as this may allow rationale for the design of effective combination therapies. Therefore, we next investigated whether targeting the HR repair pathway may be an additional approach for treatment of HTLV-I-transformed cells. The use of RI-1, up to 20 μM, had no significant effect on either the proliferation or the killing of HTLV-I-transformed MT4 cells (Figure 6C and 6D). Similar results were obtained using other HTLV-I-transformed cells (data not shown). These results may in part be explained by the fact that the HR repair pathway is already inhibited in HTLV-I-transformed cells [29] and these cells may have already adapted. As a result of a deficient HR repair pathway, HTLV-I-transformed cells are dependent on alternative DNA repair pathways. Because of intrinsic genetic instability and the presence of DDSB, identification of repair pathways needed for ATL cells may be used as an Achilles’ Heel for future therapies.

## Discussion

Although the mechanism by which HTLV-I transforms human T cells and triggers leukemia or lymphoma is not fully understood, it is clear that Tax plays a central role in this process. It is believed that Tax expression is initially needed to transform T cells but its expression may not be required to maintain the transformed phenotype, although this has not been formally demonstrated. Tax inhibits pro-apoptotic pathways and reactivates hTERT expression, thereby extending the lifespan and replicative potential of virus-infected cells. Tax has also been shown to target multiple G1/S cell cycle checkpoints to enhance proliferation of HTLV-I leukemic cells. Finally, Tax prematurely activates the anaphase promoting complex [40], inhibits nucleotide excision repair [41], and alters topoisomerases [42] and beta-polymerases [43], and the mini-chromosome maintenance, MCM2-7, helicase [30]. Consequently, Tax expression is associated with increased genomic and genetic instability.

We previously demonstrated that Tax expression is associated with an accumulation of DNA double strand breaks during S phase and that Tax inhibits the HR DNA repair pathway. In this study, we first investigated the effects of Tax on DNA replication forks. We found that Tax expression was associated with significantly shorter DNA replication forks, which also paused and stalled more often. These results suggest that Tax expressing cells have greater difficulties in replicating their DNA and progressing through complex DNA structures, which results in the accumulation of replication fork fallout and the formation of DNA double strand breaks. We also found an increase in the replication initiation rate through activation of back-up origins in Tax-expressing cells. While this could partially compensate for slower progression of the replication fork, it can also sensitize cells to accumulate additional DNA breaks.

Using BRCA1-deficient cells, deficient in the HR DNA repair pathway, we further demonstrated that Tax directly induces DNA breaks independently from its inhibitory effects on HR. In fact, we demonstrate that Tax expression stimulates NO production resulting in the formation of DNA double strand breaks. Translesion synthesis (TLS) DNA polymerases of the γ-family allow error-prone replication through damaged DNA. Among the TLS, Pol-H and Pol-K expression is mainly controlled by p53 and p21waf. It is believed that both the increase and decrease of Pol-K steady-state levels can promote a malignant phenotype. In fact, the Pol-K gene has been shown to display loss of heterozygosity in non-squamous lung carcinomas compared to adjacent normal tissue [44]. Pol-K-knockout mice have been generated and show increased spontaneous mutagenesis in the kidney, liver, and lung [45, 46]. Loss of Pol-H activity has been associated with hyper mutability and a cancer-prone syndrome known as xeroderma pigmentosum variant (XPV) [47]. Expression of TLS has never been evaluated in HTLV-I leukemic cells. Several studies have demonstrated that Tax inactivates p53 transcriptional functions through multiple pathways [48]. In addition, Tax has also been demonstrated to alter p21waf expression and functions. We therefore tested expression of Pol-H and Pol-K in HTLV-I-transformed cells, Tax-immortalized T cells and ATL cells. Overall, our results demonstrated a significant loss of both Pol-H and Pol-K gene expression in HTLV-I-transformed cells. As a result, the ability of TLS polymerases to bypass DNA lesions in HTLV-I-transformed cells may be compromised, leading to an increase in replication fork stalling and an increase in the drop off rate resulting in accumulation of DDSB and chromosome instability. Interestingly, attenuated expression of hMSH2 has been reported in ATL patients [49]. Depletion of hMSH2 impairs PCNA mono-ubiquitination and the formation of foci containing Pol-K and other TLS polymerases at stalled replication fork sites after DNA damage [50].

In response to the excessive genomic instability present in cancer cells, alterations in various DNA repair pathways are selected in most aggressive tumor cells. Because we have previously shown a shift from HR to the NHEJ DNA repair in HTLV-I leukemic cells, we investigated whether inhibition of either the NHEJ or HR repair pathway could constitute an efficient therapeutic approach. Therapeutic concentrations of the DNA-PK NHEJ inhibitor, NU7026, were effective against all HTLV-I-transformed cells and ATL cells tested, inducing a significant reduction in cell proliferation, accompanied by cell death through apoptosis. In contrast, the use of a Rad51 HR inhibitor, RI-1 had no significant effect on HTLV-I-transformed cells and ATL cells when used at concentrations sufficient to inhibit the HR pathway. Some toxicity was observed at higher concentrations but these effects may be related to inhibition of the SSA pathway or non-specific off target effects.

In cancers that are deficient in the HR repair pathway, the alternative DNA repair pathways are essential for cell viability. In fact, Poly-ADP-ribose polymerase 1 (PARP1), which plays a signaling role in the DNA Base Excision Repair pathway (BER), is critical for viability of familial breast cancer cells deficient in HR proteins BRCA1 and BRCA2. As a result, these cells are sensitive to inhibitors of PARP1. Taken together, our results suggest that identification of all alternative pathways used by HTLV-I-transformed ATL cells could open the door to new and effective combination therapies.

## Materials and methods

### Plasmids

DR-GFP and pSceI for the HR assay were provided by Dr. M. Jasin. Wild-type Tax and the Tax mutants M47 and G148V cDNA were subcloned in pCDNA3.1. pcDNA-Tax expresses the wild-type Tax, G148V expresses a Tax mutant that can activate CREB/ATF but not NF-kB, and M47 expresses a Tax mutant that can activate NF-kB but not CREB/ATF.

### *In vivo*HR DNA-repair assay

The DR-GFP reporter vector uses a modified gene for green fluorescent protein (GFP) as a recombination reporter and the I-Sce I endonuclease for the introduction of DDSBs. The DR-GFP was transfected into UWB1.289 and UWB1.289 + BRCA1 cells either with the negative-control vector or with the Sce I-expressing vector. Forty-eight hours after transfection, cells were collected and GFP + cells were counted using an LSR II flow cytometer (BD Biosciences).

### Cell culture

The HTLV-I-transformed cell line expressing Tax, MT4, and the Jurkat Tax-inducible JPX9 cells were cultured in RPMI 1640 supplemented with 10% fetal calf serum (RPMI-FCS). Expression of Tax was induced in JPX9 cells by addition of CdCl220 μM for 24 hours (referred to as JPX9 Tax+ thereafter). The UWB1.289 cell line was maintained in 50% RPMI + 50% MEGM, supplemented with 3% fetal bovine serum, and UWB1.289 + BRCA1 was maintained in the same medium. The UWB1.289 + BRCA1 cell line was a gift from Dr. Jensen (University of Kansas Medical Center). Human 293T cells were maintained in continuous culture using Dulbecco’s modified Eagle medium (DMEM) supplemented with 1% penicillin-streptomycin and 10% fetal calf serum (FCS). ATL patient-derived cell lines (KK1, KOB, ATL-55 T, SLB1, ATL-25, ATL-T, SO4 and ATL-43 T) were cultured in RPMI1640 supplemented with 20% FBS and IL-2. The ATL-derived cell line ED-40515(−), referred to as ED, was cultured in RPMI1640 supplemented with 10% FBS.

### Nitric oxide measurement

NO production from transfected cells was determined by measuring nitrite levels in the supernatants. Nitric oxide (NO) is rapidly oxidized to nitrite and nitrate, which can be used to determine NO production. The nitric oxide colorimetric assay kit (Biovision, USA) was used to measure the total nitrate/nitrite in the samples. The measurements were carried out according to the manufacturer’s protocol.

### Establishment of chromosomal non-B-DNA forming sequences 293T cell lines

The pULCtrl, pUMBR, pUMycProm plasmids [37] were kindly provided by Dr. Karen M. Vasquez, University of Texas M.D Anderson Cancer Center. pULCtrl has a 600 bp control non-B DNA sequence from the *GAPDH* gene and is not known to form non-B DNA structure. pUMycProm has 556 bp of the promoter region of the human *c-MYC* gene containing 3 Z-DNA, one H-DNA and one G-DNA forming regions. pUMBR has 520 bp from the human *BCL-2* gene major break region (MBR) containing several H-DNA forming regions. The plasmids were digested with BsaI and 10 μg of each linearized plasmid DNA was purified from agarose gel and co-transfected with 1 μg of pSIH1-puro plasmid into 293T cells. Stably transfected cells were obtained after selecting in medium containing 1 μg/ml puromycin during 10 days. The plasmids harboring non-B-DNA forming sequences contain a functional LacZ gene. To check the integration of the plasmid fragments, the genomic DNA of each transgenic cell line was isolated and subjected to PCR to evaluate the presence of the *LacZ* gene (F: 5′-CCAACTTAATCGCCTTGCGG-3′, R: 5′-GACGACAGTATCGGCCTCAG-3′) for the three stable cell lines and the *GAPDH* gene (F: 5′-GGATGCCTTTGTGGAACTGTACGG-3′, R 3′-ACAGGAACCCTCCCTCTGTTAATATC-5′) for the pULCtrl cell line; the *BCL-2-MBR* gene (F 5′-GTCATGTGCATTTCCACGTCAACA-3′, R 5′-GGATAGCAGCACAGGATTGGAT-3′) for the pUMBR cell line, and the *c-MYC* promoter region (F 5′-ATGCGTTGCTGGGTTATTTTAATCA-3′, R 5′-CGGAGATTAGCGAGAGAGGATC-3′) for the pUMycProm cell line.

### Real-time Quantitative PCR

Total RNA was extracted with TRIzol (Invitrogen), treated with DNaseI (Roche-Applied Science) and reverse-transcribed using the RNA-to-cDNA Kit (Invitrogen). cDNA was used in real-time quantitative PCR using iTaq Universal SYBR Green Supermix (Bio-Rad) on the StepOnePlus Real-Time PCR system. The following primers were used: GAPDH-F: 5′- GAAGGTGAAGGTCGGAGTC-3′, GAPDH-R: 5′- GAAGATGGTGATGGGATTTC-3′, Pol-H-F: 5′-GGTGGGTGGAATAATTGCAGTGAG-3′, PolH-R: 5′-CTGGCTTCCCGGTACTTGGTGAG-3′, Pol-K-F: 5′-GGAAGCCACGAAGGGGTCCAG-3′, Pol-K-R: 5′-GCAAATCTGTCAACCTGTAATTGTGC-3′. All cDNA samples were normalized to GAPDH expression. Fold change was calculated as the ratio of normalized expression of the target gene divided by the normalized expression of the control sample. T cells were obtained from a healthy, non-HTLV-I-infected person.

### Western blotting

Inducible expression of HTLV-I Tax in JPX-9 and the HTLV-I-transformed cell line MT4 was analyzed by Western blotting assays. Cell extracts were prepared using RIPA buffer (1% triton, 1% DOC, 1% NP40, 0.1% SDS, 0.15 M NaCl, 50 mM Tris (pH = 7.5) and protease inhibitors were resolved by 10% SDS-PAGE and transferred to a polyvinylidene difluoride (PVDF) membrane. The blot was blocked with 5% milk in PBS, and then incubated with anti-Tax antibody (NIH AIDS Reagent Program, HTLV-I Tax Hybridoma (168B17)). Reactive proteins were developed with secondary antibody conjugated to alkaline phosphatase and visualized with super signal chemiluminescent (Thermo Scientific).

### Lentivirus preparation and transduction of Tax to stable cell lines

Semi-confluent 293T cells plated in 150 mm were transfected with packaging plasmid pDNL6 (10 μg), HRCMV-Tax-ires-GFP (20 μg) and pVSV-G (10 μg) using calcium phosphate (Invitrogen) according to the manufacturer’s instructions. Supernatant was collected at 24, 48 and 72 hours and virus particles were concentrated by ultracentrifugation. Concentrated virus was used to infect stable cell lines and cells were analyzed 48 hours later.

### ϒ-H2AX staining by flow cytometry

Forty-eight hours after infection with the concentrated Tax virus, cells were harvested and fixed with 70% ethanol for 30 min, washed with PBS and permeabilized for 30 min in PBS FBS 2% Triton 0.1% (FACS buffer). Cells were resuspended in a total volume of 100 μl containing rabbit polyclonal anti-phospho-histone H2AX antibody (Cell Signaling (#2753); 1/50 dilution) and incubated 1 h under constant agitation at room temperature (RT). Samples were rinsed twice with FACS buffer and resuspended in 100 μl with the secondary antibody Alexa 488 goat anti-rabbit IgG (H + L) conjugate (molecular probes (#A11008), 1/200) for 1 h under constant agitation at RT. Samples were washed twice with FACS buffer and resuspended in PBS containing 50 μg/ml Propidium Iodide (SIGMA (P4170)) and 100 μg/ml RNAse-A before being analyzed with LSRII (Becton Dickinson). Analysis of flow cytometry data was conducted with DIVA software.

### Construction and analysis of pGEMT-Myc-tax clones

Forty-eight hours after infection with the Tax pseudotyped virus, the genomic DNA was isolated from the pUMycProm transgenic cells and subjected to PCR to amplify the *ccpeMYC* promoter region (556 bp). The PCR product was extracted from the gel (Qiagen kit) and cloned into the pGEM-T Easy vector using the manufacturer’s recommendations (Promega) and sequenced.

### DNA combing

DNA combing was performed essentially as described [4], with slight modifications. Briefly, 4.10^5^ cells were seeded for 48 h. Induction of Tax expression was done by adding CdCl2 (20 μM, 36 h). Before harvesting, cells were labelled by two successive pulses of IdU (25 μM, 15 min, MP Biomedicals) and CldU (200 μM, 15 min, MP Biomedicals). DNA fibers were extracted in agarose plugs containing 8.10^4^ cells, immediately after IdU and CldU labelling and were stretched on silanized coverslips. IdU and CldU were detected with a mouse antibody (347580; Becton Dickinson; 1/20) and a secondary antibody coupled to Alexa 546 (A21123, Molecular Probes; 1/50), and with a rat monoclonal antibody (ABC117 7513, AbCys; 1/20) and a secondary antibody coupled to Alexa 488 (A21470, Molecular Probes; 1/50). DNA molecules were counterstained with an anti-ssDNA antibody (MAB3034, Chemicon; 1/500) and an anti-mouse IgG coupled to Alexa 647 (A21241, Molecular Probes, 1/50). DNA fibers were analyzed on a Leica DM6000B microscope equipped with a CoolSNAP HQ1 4 CCD camera, with 490, 550 and 650 nm filters (Montpellier RIO Imaging facility of IGH). Data acquisition was performed with MetaMorph (Roper Scientifics). Representative images of DNA fibers were assembled from different fields of view and were processed as described [4].

### Graphs and statistical analysis

Box-and-whisker graphs were plotted with Prism v5.0 (GraphPad Software). For all graphs, the top and bottom of the box corresponds to the 25th and 75th percentile (the lower and upper quartiles, respectively) and the line near the middle of the box marks the median (50th percentile).Whiskers correspond to the 5–95 percentiles. Data not included between the whiskers are plotted as outliers (dots). Statistical analysis was performed in Prismv5.0 (GraphPad Software) using the non-parametric Mann–Whitney rank sum test.
